# PD-1抑制剂一线治疗经典霍奇金淋巴瘤致假性进展一例报告并文献复习

**DOI:** 10.3760/cma.j.issn.0253-2727.2021.12.014

**Published:** 2021-12

**Authors:** 文静 王, 杨 柯, 晶 李, 澎 刘

**Affiliations:** 复旦大学附属中山医院血液科，上海 200032 Department of Hematology, Zhongshan Hospital, Fudan University, Shanghai 200032, China

经典霍奇金淋巴瘤（cHL）是血液系统常见恶性肿瘤之一。ABVD方案（阿霉素+博来霉素+长春花碱+达卡巴嗪）化疗因其高应答率作为cHL的一线治疗多年，但化疗药物带来的严重不良反应影响患者的生活质量。近年来，复发/难治cHL对免疫检查点抑制剂PD-1单抗表现出良好的反应性和耐受性，但PD-1单抗作为免疫疗法为患者提供新机遇的同时也带来了挑战。现报道1例一线采用替雷利珠单抗（Tislelizumab）联合AVD方案（阿霉素+长春地辛+达卡巴嗪）治疗的cHL患者。此患者病程中出现典型假性进展影像学改变，经活检病理证实为假性进展，并最终获得完全缓解（CR）。

## 病例资料

患者，男，34岁，2020年3月因“反复低热伴胸闷2周”就诊于社区医院，血常规示：WBC 15.52×10^9^/L，ANC 11.27×10^9^/L；胸部CT发现纵隔占位，行抗感染治疗无效后转至我院。患者完善PET-CT示：前纵隔糖代谢异常增高的软组织密度肿块，大小约28.2 mm×21.4 mm，考虑前纵隔恶性肿瘤侵犯毗邻血管可能，伴纵隔多发淋巴结转移。患者2020年4月10日于我院胸外科行胸腔镜下标本活检，病理免疫组化染色：CD20（+），CD79α（+），CD3（70％+），CD5（70％+），CD10（+），Bcl2（+），Bcl6（20％+），CD15（大细胞+），CD30（大细胞+），Ki-67（90％+），MUM-1（部分+），PAX-5（30％+），CD19（50％+），诊断为cHL，结节硬化型（Lugano分期ⅡB期）。患者基线红细胞沉降率>30 mm/1 h，根据欧洲肿瘤内科学会（ESMO）霍奇金淋巴瘤指南[1]评定为中期患者。因纵隔肿块侵犯毗邻血管，存在放疗风险，同时患者对ABVD方案中博来霉素的远期不良反应存在顾虑，对后期生活质量有较高要求。综合考虑后于2020年5月22日开始行PD-1抑制剂联合AVD方案治疗，具体为：替雷利珠单抗200 mg，第1、15天；阿霉素25 mg/m^2^，第1、15天；长春地辛6 mg/m^2^，第1、5天；达卡巴嗪375 mg/m^2^，第1、5天。完成2个周期替雷利珠单抗+AVD方案治疗后，患者于2020年7月3日行PET-CT中期评估，结果示前纵隔肿块增大至43.8 mm×25.7 mm，伴气管腔静脉间隙淋巴结增大，余纵隔淋巴结较前缩小。患者临床症状明显缓解，无发热、胸闷、乏力症状，实验室检查见IL-8、LDH等指标平稳，考虑患者仍有获益可能，患者知情同意后继续予原方案治疗。第3个周期治疗结束时继续随访，CT示病变持续增大（47 mm×30 mm），呈轻中度强化影。最终决定2020年8月21日于CT引导下行前纵隔淋巴结穿刺活检，病理结合免疫组化提示纤维化坏死，未见明确肿瘤性病变依据，考虑假性进展，继续治疗。2020年12月10日行6个周期治疗后终期PET-CT显示：前纵隔病变呈坏死改变，缩小至41.1 mm×22.1 mm，主动脉弓旁、腔静脉间隙淋巴结均有所缩小，但仍有代谢活性，根据2014版Lugano淋巴瘤疗效评估标准[2]达部分缓解（PR）。后患者以替雷利珠单抗维持治疗，于2021年3月11日行末次PET-CT评估：纵隔病灶再次缩小至38.6 mm×19.2 mm，糖代谢活性显著降低（表1），最终评估疗效达CR。病程中患者出现1次四肢及腹股沟皮疹，无其他不良反应，总体耐受性良好。

**表1:** 患者治疗期间影像学检查结果

日期	影像学检查	纵隔血池/肝血池SUV值	前纵隔肿块		主动脉弓旁淋巴结	
			大小（mm×mm）	最大SUV值	大小（mm×mm）	最大SUV值
2020-03-10	PET-CT	–	28.2×21.4	6.1	12.1×8.8	7.2
2020-07-03	PET-CT	2.3/3.9	43.8×25.7	7.0	13.2×5.9	1.1
2020-08-14	增强CT	–	47.0×30.0	–	–	–
2020-10-07	增强CT	–	48.0×30.0	–	–	–
2020-12-10	PET-CT	1.7/3.8	41.1×22.1	4.8	5.9×3.0	3.1
2021-03-11	PET-CT	2.0/3.4	38.6×19.2	3.3	–	–

注：–：无数据

## 讨论及文献复习

cHL常以无痛性淋巴结肿大起病，好发年龄段为25～30岁和>50岁[3]，一线治疗方案为ABVD方案等多种化疗药物联用方案。但化疗药物伴随的不良反应，特别是博来霉素导致的肺损伤给年轻患者带来远期后遗症[4]，也给伴随合并症的老年患者用药带来限制，且一线化疗后仍有20％左右的患者会复发或对化疗药物不敏感[5]。目前，临床研究证实靶向T细胞免疫的PD-1/PDL-1单抗可以对抗多种肿瘤。在血液肿瘤领域，2016年纳武利尤单抗（Nivolumab）以66.3％的客观缓解率被美国FDA批准用于复发/难治cHL的挽救性治疗[6]。随后，帕博利珠单抗（Pembrolizumab）、替雷利珠单抗（Tislelizumab）也同样获批成为cHL的多线治疗方案[7]–[8]。除此之外，Ⅱ期临床试验CheckMate 205显示：PD-1单抗联合AVD方案一线治疗cHL显示出优异的疗效和耐受性[9]。德国的一项Ⅱ期临床试验采用纳武利尤单抗联合AVD方案治疗早期cHL，CR率达到94％[10]。

机制探索也发现临床使用PD-1抑制剂治疗cHL具有极大潜力。大部分cHL的R-S细胞中存在9p24.1染色体突变，该突变会导致PD-L1/PD-L2过表达。还有部分患者存在EBV感染，能够使PD-L1/PD-L2高表达[11]。目前已有多项临床试验对PD-1抑制剂一线治疗cHL进行探索（表2），其中包括NCT03004833、NCT03907488等国际多中心大样本临床试验，其研究数据有待报道。本例患者最终达CR，在一定程度上提示了一线使用PD-1抑制剂联合化疗治疗cHL的可行性。

**表2:** PD-1抑制剂一线治疗霍奇金淋巴瘤（HL）相关临床试验

试验编号	纳入人群	治疗方案	例数	首要终点	预计初步完成时间
NCT02181738[9]	初治/复发cHL	纳武利尤单抗+AVD方案	294	ORR	已发表
NCT03004833[10]	早期预后不良cHL	纳武利尤单抗+AVD方案	110	CRR	已发表
NCT03226249[12]	HL	帕博利珠单抗+AVD方案	30	CRR	已发表
NCT02758717[13]	HL老年患者	纳武利尤单抗+维布妥昔单抗	46	ORR	已发表
NCT03712202	Ⅰ～Ⅱ期cHL	纳武利尤单抗+维布妥昔单抗	264	18个月PFS期	2021-08
NCT03331341	cHL	帕博利珠单抗+AVD方案	30	不良反应发生率	2021-12
NCT03033914	cHL	纳武利尤单抗+ABVD方案	81	发生剂量限制毒性例数	2022-01
NCT04067037	ⅡB、Ⅲ、Ⅳ期cHL	卡瑞利珠单抗+AVD方案	60	ORR	2022-01
NCT03331731	HL	帕博利珠单抗	25	ORR	2022-05
NCT03907488	Ⅲ、Ⅳ期cHL	纳武利尤单抗+AVD方案+放疗；维布妥昔单抗+AVD方案+放疗	987	PFS期	2024-03
						NCT05008224	cHL	帕博利珠单抗+BEACOPP方案	140	CRR	2024-06
NCT03407144	化疗起效慢的青少年cHL	帕博利珠单抗+AVD方案；帕博利珠单抗+COPDAC-28方案	440	ORR	2027-02

注：表中数据来自ClinicalTrials.gov数据库；cHL：经典霍奇金淋巴瘤；AVD：阿霉素+长春新碱+达卡巴嗪；ABVD：阿霉素+博来霉素+长春新碱+达卡巴嗪；BEACOPP：博来霉素+依托泊苷+阿霉素+环磷酰胺+长春新碱+丙卡巴肼+泼尼松；COPDAC-28：环磷酰胺+长春新碱+泼尼松/甲泼尼龙+达卡巴嗪；ORR：客观缓解率；CRR：完全缓解率；PFS：无进展生存

当下免疫检查点抑制剂的应用如火如荼，已有不少学者观察到治疗中假性进展和延迟应答的发生[14]。接受PD-1/PD-L1单抗治疗的淋巴瘤患者也曾被报道出现病灶的自限性增大、新病灶的产生和FDG摄取的增高[15]。参考实体肿瘤相关研究，有学者推测其病理生理机制与机体免疫反应有关[16]，细胞因子调控使免疫细胞如杀伤T细胞浸润病灶，出现水肿、坏死[17]，此时肿瘤负荷往往已经极大降低。观察本例患者病程中PET-CT或CT图像可见原病灶增大、FDG摄取增高和微小新病灶产生。中期评估影像学检查中的增大病灶，依据传统Deauville标准[18]分级评分为5分，但PET-CT影像经核医学科专家阅片后考虑纵隔肿块内部为坏死灶可能性大，仅外周存在代谢活性；6个周期治疗后终期评估影像学检查显示原增大病灶回缩，提示治疗有效；后续单药维持，影像学评估病灶持续缩小，糖代谢活性显著下降，评估疗效可达CR。根据修订的Lugano淋巴瘤免疫治疗缓解标准（LYRIC）[15]，对比回顾患者治疗2个周期后PET-CT改变可进一步证实早期假性进展的诊断，具有临床参考价值。

明确为假性进展后，维持治疗能使患者持续获益。但基于cHL肿瘤微环境富集免疫细胞，以及青年患者体内未耗竭免疫细胞占上风的病理基础，假性进展的发生率并不低[19]。此类迥异于化疗反应的表现为疗效评估带来困难，目前WHO、传统Lugano标准已不能满足临床淋巴瘤的疗效评估需求，在此体系下免疫治疗假性进展极可能被误判，导致过早停药，无法实现疗效最大化。因此尽快推广和完善LYRIC影像评价分类法很有必要，恰当的病灶活检也能成为鉴别假性进展的有效方法。
